# Optimization of Turning of Inconel 625 to Improve Surface Quality After Finishing Process

**DOI:** 10.3390/ma17236009

**Published:** 2024-12-08

**Authors:** Magdalena Machno, Wojciech Zębala, Emilia Franczyk

**Affiliations:** 1 Department of Rail Vehicles and Transport, Faculty of Mechanical Engineering, Cracow University of Technology, 31-155 Cracow, Poland; 2 Department of Production Engineering, Faculty of Mechanical, Cracow University of Technology, 31-155 Cracow, Poland; wojciech.zebala@pk.edu.pl

**Keywords:** Inconel 625, turning, surface roughness, optimization, finishing process

## Abstract

The process of machining the modern engineering materials, such as nickel-based superalloys, still requires improvement. This paper focuses on comparing the turning process of Inconel 625 superalloy using three types of cutting inserts to obtain the finishing process. The influence of cutting data, such as cutting speed, feed rate, and cutting depth, on the machined surface quality, surface quality were selected. The novelty of the research, described in the article, is the optimization of the machining of Inconel 625 by using the stepwise selection of parameters. The most important issue is that the stepwise method can be used in industry, where increasingly new nickel-chromium materials with more specific strength properties are used for parts.

## 1. Introduction

Conventional machining of nickel-chromium alloys is a challenge, as these alloys are classified as extremely difficult-to-cut due to their low thermal conductivity, severe work hardening, and high chemical activity [1]. Moreover, when machining these alloys, their properties such as high strength, hardness, ductility, and tendency to harden at high temperatures make the cutting force high and the chips difficult to break. However, due to their strength properties and thermophysical properties, they are often used for parts in the aerospace, chemical, or medical industries [2,3,4].

One of the widely used alloys for parts is the Inconel 625 superalloy [5]. It is a nickel-chromium–molybdenum alloy with an addition of niobium, which, in cooperation with molybdenum, stiffens the alloy base, giving it its high strength without the need for hardening by heat treatment. Inconel 625 is characterized by [6,7]:

- good mechanical properties,
- resistance to stress corrosion cracking caused by chlorine,
- high resistance to mineral acids (such as nitric, phosphate, sulfuric, hydrochloric acid),
- good resistance to alkalis and organic acids,
- exceptional resistance to pitting, crevices, erosion, and intercrystalline corrosion.

Nickel-chromium superalloys such as Inconel 625 are widely used in the aerospace industry. The application area of these alloys has expanded to nuclear power systems, the submarine industry, the chemical and petrochemical industry, etc. In addition, this superalloy is an excellent material with high thermal resistance and the ability to retain most of its mechanical strength properties and corrosion and erosion resistance at high temperatures above 600 °C, making it attractive for demanding applications [6]. Also, the tensile and creep strength tests of the Inconel 625 superalloy showed its effective use in steam turbine parts. Its use in steam turbine castings can improve the thermal efficiency of steam turbines. Another potential application of the Inconel 625 superalloy is as heat exchanger tubes in microturbines and advanced aircraft engines [7].

Due to the properties and behavior of nickel–chromium alloys, it is difficult to select an appropriately durable tool during machining. During the machining of these alloys, the tools wear out quickly, generating additional costs in the technological process of manufacturing parts [3,8]. The costs increase particularly when a finished surface is needed. In addition, the selection of cutting parameters (cutting speed, feed per revolution, depth of cut) is a challenge for these alloys. Therefore, cutting parameter optimization methods are most often developed in order to efficiently process [9,10]. Methods using neural networks are also used to optimize turning parameters [11,12]. 

There are many scientific works in the literature describing the above challenges in the cutting process of modern nickel–chromium alloys [1]. During the machining process, the interaction between the tool and the workpiece causes severe plastic deformation in the machining area and increased tool wear. The difficulties in machinability of Ni-based superalloys cause a challenge in selecting effective cutting tools [13]. Currently, there are many methods for analyzing the effects of machining process parameters on process efficiency or machined surface roughness. Among the data analysis methods used in machining processes, the following methods are distinguished: Taguchi Grey and Technique for Order Preference by Similarity to Ideal Solution (TOPSIS) [14] or artificial neural network (ANN)-based models [15]. These publications also indicate that it is worth comparing the above-mentioned methods for developing mathematical models. Also, many works also focus on the optimization of cutting process parameters such as cutting speed, feed per revolution, cutting depth, surface roughness or forces occurring in the machining area [16,17]. Liu at al. [2] analyzed the effect of cutting parameters on the surface roughness and wear of the cutting insert flank, taking into account the wear map. The analysis was performed to determine the optimal parameters. The machining was performed using a PVD-TiAlN-coated carbide tool under conventional cooling conditions. The optimal cutting parameters were selected: cutting speed—60 m/min, feed per revolution—0.1 mm/rev, and cutting depth—0.5 mm. The authors emphasize that for the Inconel 625 superalloy, tool wear is a serious problem in the machining process. The paper proposes a tool life reliability index at optimal machining parameters. In the paper, however, [9], an attempt was also made to find the optimal parameters of the Inconel 625 turning process. The influence of cutting speed, feed rate, depth of cut on cutting force, surface roughness, material removal rate was investigated in order to find the optimal values of the process parameters. From the conducted analysis and the adopted method of selecting parameters, the following values were selected as the most optimal: 60 m/min for cutting speed, 0.3 mm/rev for feed rate, 0.25 mm for depth of cut. Another method of optimizing the Inconel 625 turning parameters is presented in [11]. The research paper presents a developed mathematical model of surface roughness using the Taguchi method and regression analysis. Turning tests were performed using cryogenically treated tungsten carbide inserts. The developed roughness prediction models are very useful in the case of optimizing the machining of difficult machine materials such as Inconel 625. However, in the paper [3], Inconel 718 turning is studied using different cutting inserts such as commercial PCBN tools with low CBN content from different manufacturers: CBN 170 (Seco), KB5625 (Kennametal), MB8025 (Mitsubishi), and 7015 (Sandvik). The cutting inserts used had a cutting edge configuration suitable for finishing turning. The PCBN 7015 tool showed the highest cutting edge durability, followed by CBN170. The best cutting parameters for cutting edge durability corresponded to a cutting speed of 250 m/min and a feed rate of 0.15 mm/rev. Research results and their analysis in [18] showed that the CBN content in the tool strongly affects the tool performance. The insert based on 45% CBN showed the best performance in finishing operations of Inconel 718.

In addition, new methods for selecting the most promising parameters are being developed to analyze the results of the turning process. An interesting approach to data analysis and selection of the most promising parameters is presented in [19]. In this work, surface roughness was analyzed theoretically and experimentally. The selection of the obtained roughness of the turned surface was made using two methods. In the first step, equations were derived that determine the analyzed roughness parameters, and in the second step, experiments were carried out to further analyze and validate the results of mathematical models. A comparison of experimental and theoretical results showed that the derived equations allow for the prediction of the arithmetic mean roughness and the average roughness depth. This is important when developing technological processes for manufacturing parts. Also, a novel cutting technology for hardened, rotationally symmetric parts is presented in [20]. This novel technique is called rotational turning, and it combines hard turning and circular milling. In this paper, an analytical model is developed to describe the coupling parameters between the tool and the workpiece. The model shows that the virtual tool corner radius in rotational turning, which takes place during the cutting process, is more than 50 times larger than in the state-of-the-art hard turning. Furthermore, it is shown in this paper that the minimum achievable surface roughness is not limited by the feed rate, but also by other effects such as cutting edge waviness.

When analyzing the results of the turning process, for manufacturing technology, the most important thing is the quality of the machined surface, i.e., its roughness parameters. Hence, in scientific works, one of the most common result parameters after the turning process and at the same time the most important from the technological point of view, is the surface roughness Ra and Rz [19,21,22].

The above examples from the literature show that there is still a need to optimize the parameters of the turning process of nickel–chromium alloys and it is difficult to use appropriate tools available on the market for effective machining of, e.g., Inconel 625 or Inconel 718. However, in the aviation industry there is an increasing usage of nickel–chromium superalloys for manufactured parts due to their favorable mechanical and thermophysical properties [23,24]. 

The analysis of research results presented in scientific articles shows that methods or data analysis systems that facilitate the optimization and selection of parameter values to obtain the most promising ones still require development. The use of superalloys on parts is still a challenge for the development of effective manufacturing processes. Effective methods are still sought to effectively select the right values of machining parameters. There is still a gap in this matter. Therefore, in this article we decided to conduct experimental tests of turning Inconel 625. The main goal of this work is to select the turning parameters of this superalloy to obtain a surface after finishing.

Accordingly, in this paper, the focus of the turning tests was on the machining of Inconel 625 and checking its turning for several cutting inserts. Within the paper, the optimization of turning parameters was performed in order to obtain the technologically required surface quality (roughness Ra, Rz). The paper analyzed the influence of turning parameters (cutting speed, feed per revolution, depth of cut) and the influence of the type of cutting insert (due to the corner radius) on the roughness of the machined surface. The selection of the most promising parameters and the type of insert was developed in three stages. During the analysis, attention was also paid to the process efficiency and obtaining a finished surface. As a novelty, the paper determined the optimization of the turning parameters of Inconel 625, using a three-stage stepwise method of selecting the tool and turning parameters. The main objective of this work is to find the values of the turning tool and cutting tool parameters that give the most satisfactory surface quality results, i.e., provide the surface after finishing. The innovation of this work is the algorithm, which includes comprehensively presenting the method of selecting the most promising parameters in order to obtain the best surface roughness. Its complexity is based on the stepwise selection of cutting parameters, which can be used in industry.

## 2. Materials and Methods

The turning tests were performed at the Cracow University of Technology. A shaft made of nickel–chromium superalloy—Inconel 625, with a diameter of 50 mm—was selected as the machined material for the tests. These types of superalloys are classified as difficult-to-machine, and optimal parameters for their turning are still being selected. Therefore, this material was selected for the turning tests. Table 1 presents the chemical composition of Inconel 625, while Table 2 presents its selected mechanical and thermo-physical properties.

The experimental turning tests were carried out according to the Taguchi plan with three repetitions for each test. For this plan, three sets of tests were performed. Each set of tests was performed using a different cutting tool—a cutting insert from the SECO Producer. The individual cutting inserts had the following catalog designations [26]:

- CNMG120404-MF1, CP200—cutting insert No. 1,
- CNMG120404-MF1, TH1000—cutting insert No. 2,
- CNMG120408-MF4, TS2500—cutting insert No. 3.

**Figure 1:**
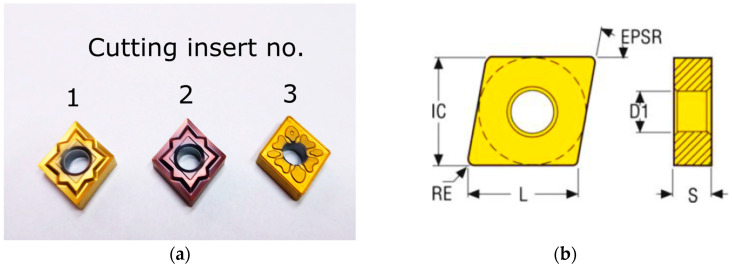
(**a**) Cutting inserts used in experimental test; (**b**) cutting insert of geometry.

Three turning tools were used for the tests, which differed in the corner radius, chip breaker and presence of a coating. Tools 1 and 3 have a protective coating, tool 2 is not coated. The geometry of the cutting inserts is shown in Table 3. The tools have been assigned numbers 1, 2, and 3. Figure 1a shows the cutting inserts. Table 3 presents selected specifications of the cutting inserts used. However, in Figure 1b there are symbols included in Table 3.

**Figure 2:**
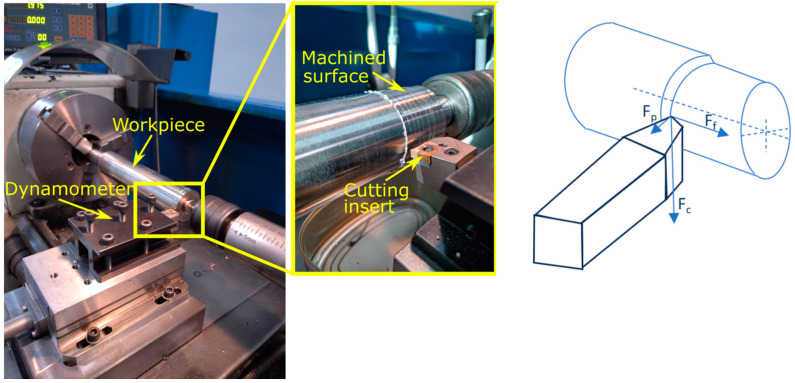
Experimental test stand and marked directions the components of the total cutting force.

**Figure 3:**
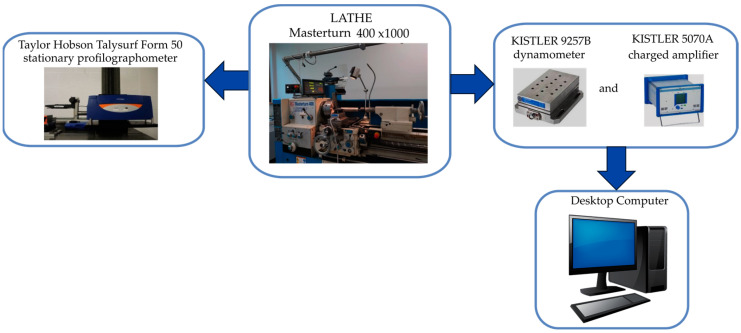
The scheme of the experimental test.

The cutting insert was mounted in a PCLNR 2020K-12 holder. The turning tool was mounted on a lathe with clearance angles of 95° and a side cutting edge angle of 6°.The test stand is shown in Figure 2. The dry turning tests were performed on a Knuth Masterturn 400 × 1000 lathe (see Table 4 for a characteristic of the lathe). During turning, the components of the total cutting force (*F_c_*—cutting force, *F_f_* —feed force, *F_p_*—radial force) were measured using the KISTLER 9257B piezoelectric dynamometer. The components of the total cutting force were measured to compare their values for the three types of inserts used during the tests. However, the components of the total cutting force were not an analyzed factor influencing the roughness parameters. The dynamometer was connected to the KISTLER 5070A charge amplifier. The forces in the turning process were recorded and observed on a computer station connected to the dynamometer. In Figure 3, the scheme of experimental test is presented. After turning, the obtain surface was measured by using a Taylor Hobson Talysurf Intra 50 stationary profilographometer (Taylor Hobson, Leicester, UK). The two-dimensional (2D) surface roughness for Ra and Rz parameters was measured. The roughness measurement was performed using a tip with a rounding radius of 2 µm. A soft wave profile filter *λ_c_* = 0.8 mm was used for the measurements. The roughness measurements Ra and Rz were performed in accordance with ISO 4287. For one surface, three measurements (repetitions) were performed on a section of 4 mm in length. The average value of the three measured Ra and Rz values for a given machined surface was taken into account for the analysis.

The process parameters selected for analysis were cutting speed—*v_c_*, feed per revolution—*f* and depth of cut—*a_p_*. In addition, the shape and type of the obtained chips were analyzed. Table 5 presents the analyzed levels of turning parameter values, while Table 6 presents the adopted Taguchi research plan and the resulting parameters.

The aim of the work was to select the most promising parameter values for turning Inconel 625 in order to obtain the lowest possible surface roughness Ra and Rz, which would best ensure the surface after finishing. The work proposed a step-by-step method for selecting parameters in three stages. First, all obtained results were analyzed. In the first stage of selection, the type of tool was analyzed. In the second stage, the influence of cutting depth was studied. In the third stage, the lowest values of roughness Ra and Rz were selected for the chosen parameters. Analysis of Variance (ANOVA) was performed for the most promising parameters of the turning process with the use of the Minitab statistical software 22 (Minitab LLC., State College, PA, USA). The main aim of this work was optimization of all parameters to obtain surface matching the finished workpiece characteristic.

## 3. Results and Discussion

### 3.1. The Surface Roughness Results

**Figure 4:**
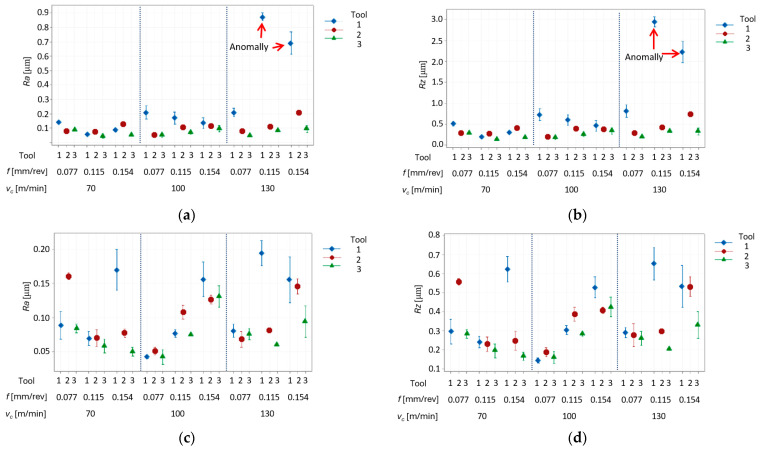
Results of surface roughness vs. feed rate (*f*), cutting speed (*v_c_*), and used tool No. 1, No. 2, and No. 3: (**a**) Ra and (**b**) Rz—for depth of cut *a_p_* = 0.1 mm; (**c**) Ra and (**d**) Rz—for depth of cut *a_p_* = 0.5 mm.

In the first stage, the roughness parameters, Ra and Rz, were analyzed for all surfaces. Figure 4a–d show the results for different cutting inserts, feed rates, depths of cut and cutting speeds. Each result is the arithmetic mean of three measurements. At this stage, the influence of turning parameters and the influence of three cutting inserts on surface roughness were analyzed.

It is observed that the minimum roughness value was 0.05 μm for Ra and 0.02 μm for Rz (for *v_c_* =70 m/min, *f* = 0.154 mm/rev, *a_p_* = 0.1 mm). However, the maximum Ra values were 0.86 μm and Rz values 2.88 μm for *v_c_* = 130 m/min, *f* = 0.115 mm/rev, *a_p_* = 0.1 mm. An important conclusion is that, in accordance with the principles of designing the manufacturing technological processes, these values are for the surface quality after finish machining.

**Figure 5:**
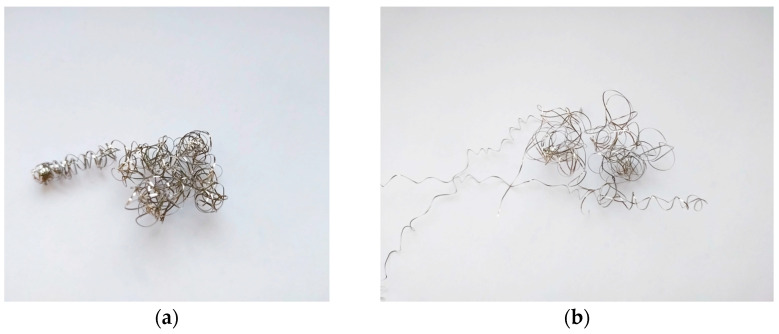
Chips after turning with the parameters: (**a**) *v_c_* = 130 m/min, *f* = 0.115 mm/rev, *a_p_* = 0.1 mm; (**b**) *v_c_* = 100 m/min, *f* = 0.077 mm/rev, *a_p_* = 0.1 mm.

On the other hand, for a depth of cut of 0.1 mm, again tool No. 1, and a cutting speed of 130 m/min, and feed rates of 0.115 mm/rev and 0.154 mm/rev (Figure 4a,b), the obtained roughness values were significantly higher. The chips were, again, long and tangled in these tests. Such chips could damage the machined surface during the turning, which may explain the higher values of Ra and Rz. Moreover, there were tests where the chip coiled itself strongly near the machined surface, damaging it. These tests were classified as anomalies. The chip appearance for the anomaly tests using tool No. 1 is shown in Figure 5a. However, for comparison, Figure 5b shows the chip for turning, which gave a better surface quality (Ra about 0.04 µm) for this tool.

The analysis of the roughness results in Figure 4a–d shows that tool No. 1 gives higher roughness values compared to tool No. 2 and tool No. 3. Hence, at this stage it was decided to reject the surface roughness results for tool No. 1. In the following part of the analysis, the results after turning with tool No. 2 and tool No. 3 are shown.

**Figure 6:**
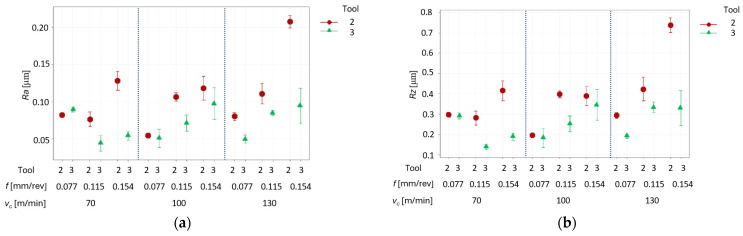

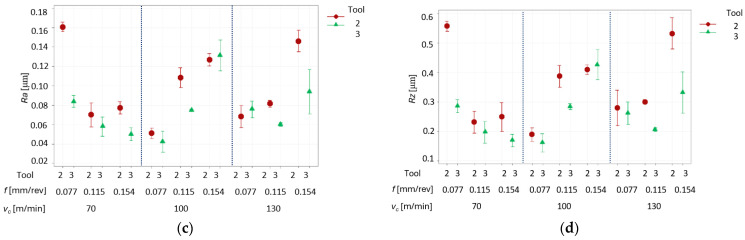
Results of surface roughness vs. feed rate (*f*), cutting speed (*v_c_*), and used tools No. 2 and No. 3: (**a**) Ra and (**b**) Rz—for depth of cut *a_p_* = 0.1 mm; (**c**) Ra and (**d**) Rz—for depth of cut *a_p_* = 0.5 mm.

After processing the data set and after removing the results for tool No. 1, the graphs with results for tools No. 2 and No. 3 are presented in Figure 6a–d.

Analyzing the roughness results using tools No. 2 and No. 3, it can be assumed that there is a larger group of turning parameter sets to obtain similarly lower Ra and Rz values for tool No. 3 at the applied depth of cut of 0.1 mm. This gives wider possibilities to find the most promising process parameters faster.

At that stage of selecting the most promising parameters, the authors chose the depth of cut 0.1 m and the coated cutting insert No. 3 with a corner radius of 0.8 mm. This conforms to the results of studies to date. The lesser depth of cut ensures a better quality of the machined surface, and the larger corner radius gives a smaller roughness of the machined surface. It can be inferred that with such a trend in results, the cutting inserts and parameters were selected correctly. The further analysis focused on the most promising results at that stage. The ANOVA was performed for these results, and it formed the basis for analyzing the impact of the cutting speed and feed rate per revolution on the roughness Ra and Rz and on the forces in the machining area.

### 3.2. The Cutting Forces Results

**Figure 7:**
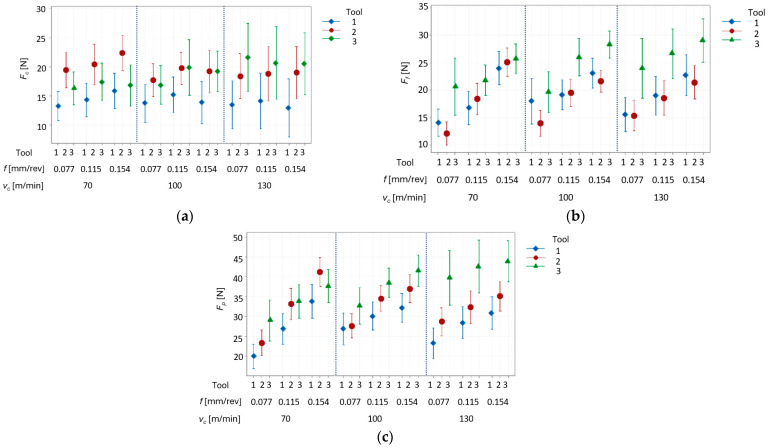
Cutting forces measurements: (**a**) *F_c_*; (**b**) *F_f_*; (**c**) *F_p_*, for vs. feed rate, cutting speed and depth of cut 0.1 mm, with three tools used.

Additionally, during the experimental tests, the component of the total cutting force was measured. However, this parameter was not a factor in the final result. The forces were measured to compare their values for the individual types of cutting inserts used. This force consists of forces such as: *F_c_*—cutting force; *F_f_*—feed force; and *F_p_*—radial force. As the previous analysis showed that the lesser Ra and Rz values were obtained for *a_p_* = 0.1 mm, in the next step the cutting forces were analyzed only for one depth of cut—0.1 mm. The results of the measured forces during the experimental tests are presented in Figure 7a–c.

The results above strongly indicate a significant impact of the feed rate per revolution on the forces present during the turning. For feed force *F_f_* and radial force *F_p_*, the increase in values is observed when the feed rate increases (Figure 6b,c). This results from the fact that those forces are related to the feed rate per revolution, hence this parameter has the strongest impact on the forces. The increase in the feed rate also increases the chip height, which leads to lower specific cutting force. This explains, why the cutting force remains nearly the same. In addition, the higher feed rate values generate larger roughness of the machined surface which indicates presence of the greater forces during the turning. The increase in the cutting forces values for the higher feed rate per revolution is absolutely normal here. 

### 3.3. Analysis of the Impact of the Turning Parameters on Ra and Rz Based on Earlier Analysis

In the next stage of the study, the statistical analysis (ANOVA) was performed using the Minitab statistical software 22 (Minitab LLC., State College, PA, USA). The analysis was performed for the selected results of tests carried out with tool No. 3 and the depth of cut of 0.1 mm. The previous analysis of results showed that for these parameters and this tool, the roughness values Ra and Rz were the smallest.

The ANOVA was performed for Ra and Rz and is shown in Table 7 and Table 8. These tables contain markings: DF is degrees of freedom, Seq SS is sums of squares, Adj SS is the adjusted sums of squares, and Adj MS is the adjusted mean squares.

The regression Equations (1) and (2), performed in the Minitab software are listed below for Ra and Rz. The values *xi* of the *v_c_xi_* and *f__xi_* coefficients in the subscript correspond to the values of these parameters at the plan level.

Ra = 0.07054 − 0.00824 v_c_70_ + 0.00253 v_c_100_ + 0.00571 v_c_130_ − 0.00741 f__0.077_ − 0.00398 f__0.115_ + 0.01140 f__0.154_ + 0.03448 v_c_70_·f__0.077_ − 0.01469 v_c_70_·f__0.115_ − 0.01980 v_c_70_·f__0.154_ − 0.01512 v_c_100_·f__0.077_ + 0.00225 v_c_100_·f__0.115_ + 0.01287 v_c_100_·f__0.154_ − 0.01936 v_c_130_·f__0.077_ + 0.01244 v_c_130_·f__0.115_ + 0.0693 v_c_130_·f__0.154_ (1)

Rz = 0.2500 − 0.0447 v_c_70_ + 0.0102 v_c_100_ +0.0345 v_c_130_ − 0.0286 f__0.077_ − 0.0087 f__0.115_ + 0.0373 f__0.154_ + 0.1136 v_c_70_ ·f__0.077_ + 0.0583 v_c_70_·f__0.115_ − 0.0553 v_c_70_·f__0.154_ − 0.0486 v_c_100_·f__0.077_ + 0.0011 v_c_100_·f__0.115_ + 0.0475 v_c_100_·f__0.154_ − 0.0650 v_c_130_·f__0.077_ + 0.0571 v_c_130_·f__0.115_ + 0.0078 v_c_130_·f__0.154_ (2)

**Figure 8:**
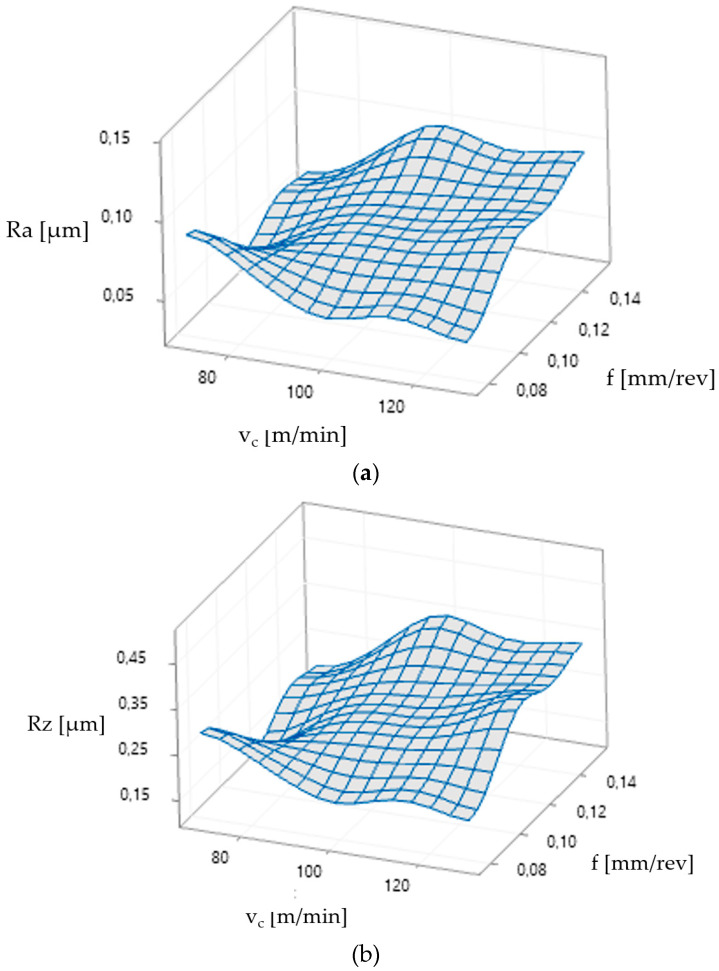
The surface roughness and interval plot for: Ra (**a**); Rz (**b**).

In the final step, plots were generated based on the regression equations and are shown in Figure 8a,b.

The obtained Ra and Rz roughness values indicate the trends characteristic for machining. The increased (*v_c_*) and lower feed rate per revolution (*f*) ensure a better surface quality (lesser values for Ra and Rz). It can be assumed that the process parameters and the cutting insert used allowed obtaining the surface roughness of the machined surfaces below 0.1 μm for Ra and below 0.4 μm for Rz. This confirms that the turning parameters and the cutting insert for machining of Inconel 625 were selected correctly. 

**Figure 9:**
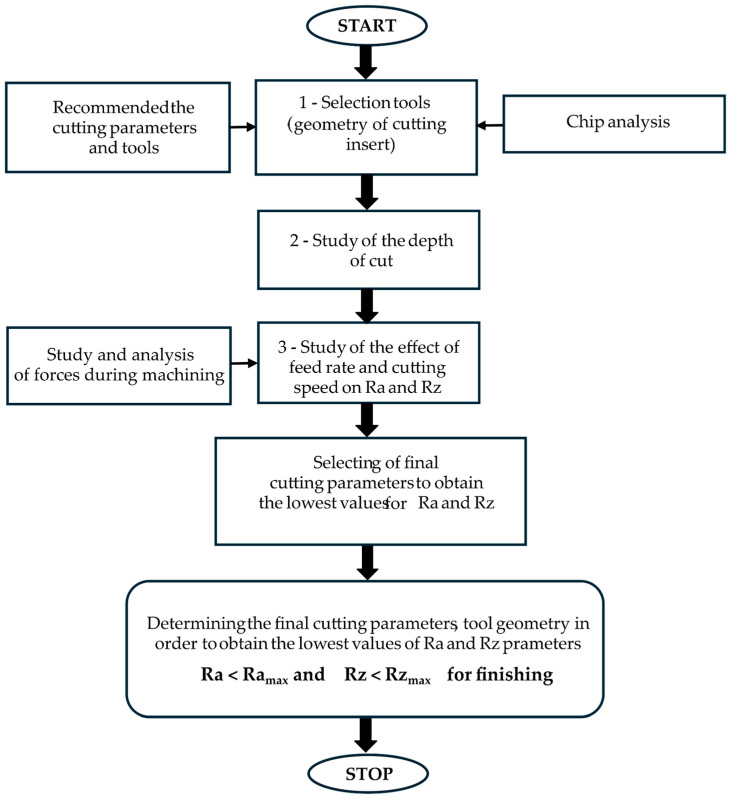
The applied algorithm for stepwise selection of parameters.

The developed algorithm (Figure 9) is presented below, showing the stepwise selection of the most promising parameters adopted in the work. The most promising parameters were those that provided the roughness values Ra and Rz for the surface after finishing.

Based on the applied multistage selection process of parameters giving the surface after finish machining, the selected parameters were applied for the depth of cut *a_p_* = 0.1 mm and the cutting tool No. 3. The purpose of the study was to find cutting parameters and tool geometry to obtain the appropriate Ra and Rz quality parameters of the machined surface of 0.1 μm and below 0.4 μm, respectively. The minimum values of Ra < 0.05 µm and Rz < 0.15 µm were obtained for the process parameters: a cutting speed of 70 m/min, a feed per revolution of 0.115 mm/rev, and a cutting depth of 0.1 mm, using tool No. 3. 

Such roughness values are classified as surface after finish machining. The selection of process parameters and the stepwise selection method used in this work can accelerate the selection of the most promising parameters in the industry. No additional tools will be necessary for the rough machining and the finish machining. The results of the analysis also confirm that the applied method for selection of the most promising process parameters was correct.

## 4. Conclusions

In this paper, an algorithm was proposed, which describes a step-by-step method for selecting the most promising parameters for the turning process of the Inconel 625 superalloy. The applied step-by-step parameter selection method allowed for selecting the most promising parameters, ensuring the surface after the finishing process (obtaining a machined surface with a Ra value below 0.1 μm and Rz below 0.4 μm). Based on the analysis of the results, the following conclusions can be drawn:

- The lowest values of Ra < 0.05 μm and Rz < 0.15 μm were obtained for the process parameters: cutting speed of 70 m/min, feed per revolution of 0.115 mm/rev, and depth of cut of 0.1 mm, with a cutting insert with a corner radius of 0.8 mm. A surface with such Ra and Rz values provides a surface after finishing.
- Recording the cutting force values during all tests showed that the Fc component has very similar values for all cutting speed and feed values. However, the Ff and Fp components depend on the applied feed—the higher the feed, the higher the forces (increase up to 70%). On the other hand, the cutting speed has a small effect, amounting to only a dozen or so percent.
- The chip breaker that is present in tool No. 3 (with a corner radius of 0.8 mm) allowed for better chip control during machining. However, in the case of tools 1 and 2 (both with a corner radius of 0.4 mm), the chips did not always flow into the chip breaker zone, which contributed to the formation of tangled chips. This phenomenon generates higher roughness values and does not give repeatable results.

The applied method of the stepwise selection of parameters and cutting insert has proven effective in the analysis of experimental test results. It is assumed that such an approach in the development of technological processes can be used in the aviation industry, for example. This method can also provide a more accurate selection of the values of appropriate parameters compared to the systems used in programs from the beginning of the analysis. In the case of a large amount of data, this method may be more time-consuming. However, the selection of parameters for turning nickel–chromium superalloys is a challenge in the manufacturing industry. Therefore, a stepwise selection of parameters and later application of programs and systems (e.g., Analysis of Variance or Response Surface Methodology) to already partially selected parameter values will give a better effect in some cases. In addition, it is worth emphasizing that the use of various statistical methods allows for faster data optimization. And as we know, this allows for lower costs, resource conservation, and increased availability on the market. 

## Figures and Tables

**Table 1:** Chemical composition of Inconel 625.

Element	Ni	Cr	Mo	Fe	Nb	Co	Si	Mn	Ti	Al	C
wt. [%]	Min. 58	20–23	8–10	5	3.2–4.2	1	0.5	0.5	0.4	0.4	0.1

**Table 2:** Important properties of Inconel 625 [25].

Property	Value
Density [g/cm^3^]	8.4
Tensile strength [MPa]	1050
Elastic modulus [GPa]	205
Hardness [HB]	320
Thermal conductivity [W m^−1^ K^−1^]	10
Poisson’s ratio	0.31
Specific heat [J kg^−1^ K^−1^]	410
Elongation to break [%]	25–30

**Table 3:** Geometry and specifications of the cutting inserts for Tool 1, 2, and 3 [26].

	Cutting Insert no.		
	1	2	3
Corner radius, RE [mm]	0.4		0.8
Insert included angle, EPSR [o]	80		
Cutting edge length [mm]	12.9		
Fixing hole diameter, D1 [mm]	5.1		
Inscribed circle diameter, IC [mm]	12.7		
Insert thickness, S [mm]	4.76		
Chip breaker	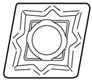	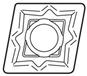	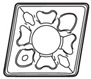

**Table 4:** The characteristic of the Knuth Masterturn 400 × 1000 lathe.

Characteristic	Value
Turn diameter, mm	400
Turn length, mm	1000
Speed range, rpm	50–950/160–3000
Total power, kW	7.5

**Table 5:** Selected cutting parameter for turning process.

Parameter	Levels Value			
	1	2		3
Cutting speed—*v_c_*, m/min	70	100		130
Feed rate—*f*, mm/rev	0.077	0.115		0.154
	**1**		**2**	
Depth of cut—*a_p_*, mm	0.1		0.5	

**Table 6:** Test plan and resulting parameters.

	Turning Parameter			Resulting Parameters	
Test No.	*v_c_* [m/min]	*f* [mm/rev]	*a_p_* [mm]	Ra [µm]	Rz [µm]
1	70	0.077	0.1	0.105	0.402
2	70	0.115	0.1	0.067	0.244
3	70	0,154	0.1	0.094	0.322
4	100	0.077	0.1	0.142	0.472
5	100	0.115	0.1	0.153	0.534
6	100	0.154	0.1	0.140	0.483
7	130	0.077	0.1	0.121	0.500
8	130	0.115	0.1	0.340	1.147
9	130	0.154	0.1	0.361	1.147
10	70	0.077	0.5	0.118	0.408
11	70	0.115	0.5	0.090	0.286
12	70	0.154	0.5	0.112	0.347
13	100	0.077	0.5	0.037	0.156
14	100	0.115	0.5	0.090	0.330
15	100	0.154	0.5	0.160	0.517
16	130	0.077	0.5	0.074	0.267
17	130	0.115	0.5	0.102	0.362
18	130	0.154	0.5	0.126	0.450

**Table 7:** The results of ANOVA—Ra.

Source	DF	Adj SS	Adj MS	F-Value	*p*-Value
*v_c_*	2	0.000961	0.000481	0.98	0.394
*f*	2	0.001806	0.000903	1.84	0.187
*v_c_·f*	4	0.008320	0.002080	4.24	0.014
Error	18	0.008829	0.000491		
Total	26	0.019917			

**Table 8:** The Results of ANOVA—Rz.

Source	DF	Adj SS	Adj MS	F-Value	*p*-Value
*v_c_*	2	0.02964	0.014822	2.43	0.117
*f*	2	0.02056	0.010279	1.68	0.214
*v_c_·f*	4	0.09458	0.023645	3.87	0.019
Error	18	0.10997	0.006109		
Total	26	0.25475			

## Data Availability

The original contributions presented in the study are included in the article; further inquiries can be directed to the corresponding author.
